# Independent and joint associations of a body shape index and cardiorespiratory fitness with executive function in adolescents: a cross-sectional study in China

**DOI:** 10.3389/fnut.2026.1739948

**Published:** 2026-02-06

**Authors:** Cunjian Bi, Xiaokang Ran, Feng Zhang, Xinming Ye, Xiaojian Yin, Mengmeng Zhang, Pengwei Sun

**Affiliations:** 1 School of Physical Education, Chizhou University, Chizhou, China; 2 ECUST-Chengxin Research Institute of Health Science and Engineering, East China University of Science and Technology, Shanghai, China; 3 School of Sports Science and Engineering, East China University of Science and Technology, Shanghai, China; 4 Department of Physical Education, Shanghai Institute of Technology, Shanghai, China,; 5 School of Physical Education, Xinjiang Normal University, Urumqi, Xinjiang, China

**Keywords:** a body shape index, adolescents, association, cardiorespiratory fitness, executive function

## Abstract

**Background:**

Executive function plays a crucial role in adolescent development and future adult achievement. However, previous research on the associations between a body shape index (ABSI), cardiorespiratory fitness, and executive function has been limited. No studies have yet been conducted using nationally representative samples. This study aims to analyze the independent and joint associations among ABSI, cardiorespiratory fitness, with executive function using a national sample, thereby providing support for enhancing and intervening in executive function among Chinese adolescents.

**Methods:**

From 2022 to 2023, a three-stage stratified cluster sampling method was employed to randomly select 8,621 adolescents aged 13–18 years across nine regions in China for a cross-sectional assessment of ABSI, cardiorespiratory fitness, and executive function. The independent and joint associations between ABSI, cardiorespiratory fitness, with executive function were analyzed using chi-square tests, *t*-tests, one-way ANOVA, binary logistic regression, and generalized linear model binary logistic regression.

**Results:**

The VO_2max_ of Chinese adolescents is (41.12 ± 5.08) mL/kg/min; the ABSI is (0.0615 ± 0.0240). The inhibit control function reaction time, the refreshing memory function reaction time, and the switching flexibility function reaction time are (13.69 ± 11.89) ms, (1055.05 ± 354.69) ms, and (320.32 ± 182.66) ms, respectively. Compared across the ABSI groups (Q1, Q2, Q3, Q4), statistically significant differences were observed in reaction times for the adolescent inhibit control function, refreshing memory function, and switching flexibility function (*χ*^2^ = 72.642, 184.613, 2826.466, *p <* 0.001). Similarly, statistically significant differences were observed in VO_2max_ between Q1, Q2, Q3, and Q4 groups (*χ*^2^ = 54.539, 47.333, 42.127, *p <* 0.001). Generalized linear model binary logistic regression analysis revealed that compared with the group with ABSI is Q1 and VO_2max_ is Q4, the group with ABSI is Q3 and VO_2max_ is Q1 exhibited a higher risk of developing: refreshing memory function dysfunction (OR = 6.20, 95% CI: 4.42–8.70), and switching flexibility function dysfunction (OR = 4.76, 95% CI: 3.48–6.52; *p <* 0.001).

**Conclusion:**

There are independent and joint associations between ABSI, cardiorespiratory fitness and executive function among Chinese adolescents. Effectively controlling the increase in ABSI and improving cardiorespiratory fitness levels play a positive role in promoting executive function in adolescents.

## 1 Introduction

Executive function, as a crucial control mechanism of the brain, plays a vital role in adolescents’ emotional regulation, attention focus, planning abilities, behavioral control, memory capacity, and cognitive capabilities (1). The execution function mainly includes three core functions: the inhibit control function, the refreshing memory function, and the switching flexibility function. Research indicates that there is a strong correlation between adolescents’ executive function levels and their academic performance. Higher executive function levels are closely associated with improved mathematics and English scores (2). Research also indicates that there is a close association between adolescents’ executive function levels and their mental health status (3). Research has found that individuals with lower levels of executive function have a higher risk of developing depression and anxiety compared to those with higher levels of executive function (4). This highlights the critical importance of adolescents’ executive function levels for their healthy development, which warrants attention and emphasis. Research indicates that adolescents’ executive function levels are influenced by a combination of factors, including physical fitness, exercise habits, muscle strength, dietary behaviors, family environment, sleep quality, brain structure, and natural surroundings (5–9). However, in recent years, research on adolescents’ executive function levels has shown an upward trend. Previous studies have primarily focused on the relationship between physical exercise, sleep, dietary behaviors, and executive function in adolescents, with limited research specifically targeting the Chinese adolescent population (10, 11). Additionally, no studies have yet been identified that examine the association between ABSI, cardiorespiratory fitness, and executive function among Chinese adolescents using a national sample.

As living standards improve, levels of physical activity continue to decline, leading to reduced cardiorespiratory fitness among adolescents and a persistent rise in obesity rates (12). A survey of Chinese adolescents reveals that the rates of overweight and obesity among youth have risen from 1.0 and 0.1% in 1985 to 14.0 and 6.4% in 2014, respectively. This trend continues to escalate, posing a serious threat to the physical and mental health of adolescents (13). ABSI serves as a comprehensive indicator reflecting participants’ body composition. Calculated using a fixed formula based on BMI and waist circumference, this metric effectively reflects abdominal fat levels in the body. This indicator was developed by Krakauer et al. and has gradually gained widespread adoption among scholars (14–16). Previous studies have primarily employed indicators such as BMI and WC to assess adolescent obesity levels. While these metrics offer simplicity and convenience in testing, they also present certain limitations, failing to comprehensively reflect abdominal obesity in adolescents (17). Research has found that BMI, waist circumference (WC), and waist-to-height ratio (WHtR) provide more accurate predictions of all-cause mortality risk and hypertension in adolescents compared to the ABSI index (18, 19). However, the results were not entirely consistent. Another study found that WC and BRI were the two best predictors for all chronic metabolic diseases except metabolic syndrome, while ABSI was the worst predictor (20). Unfortunately, there is currently a lack of research using the ABSI metric to evaluate the relationship between body composition and other health factors among Chinese adolescents. To date, no studies have been identified examining the association between ABSI and executive function in Chinese adolescents.

Cardiopulmonary fitness, as a core component of adolescents’ physical health, significantly impacts academic performance and future achievements (21). Research indicates that individuals with lower cardiorespiratory fitness exhibit relatively poorer academic performance compared to those with higher cardiorespiratory fitness (22). Research has also found that adolescents with higher cardiorespiratory fitness have a lower risk of developing mental health issues compared to those with lower cardiorespiratory fitness (23). This demonstrates that adolescents’ cardiorespiratory fitness levels are closely linked to their physical and mental health development. However, multiple studies in recent years have confirmed that as socioeconomic development and informatization advance, adolescents’ cardiorespiratory fitness levels show a significant downward trend, leading to the emergence of a series of health issues (24, 25). Therefore, effectively monitoring adolescents’ cardiorespiratory fitness levels is crucial for improving their health status. Notably, while executive function serves as a key factor in adolescent development, past research has rarely examined the relationship between cardiorespiratory fitness and executive function. Limited studies indicate a strong association between adolescents’ cardiorespiratory fitness and executive function reaction times, with higher fitness levels correlating with superior executive function performance in adolescents (26). However, past research has also yielded inconsistent findings. A study of secondary schools in southern Greenland revealed that changes in cardiorespiratory fitness did not effectively improve executive function or mental health, and these effects were not influenced by sex or socioeconomic status (27). Therefore, it is necessary to further explore the relationship between cardiorespiratory fitness and its executive functions in the future.

Cardiopulmonary fitness, body composition, and executive function serve as crucial indicators of physical and cognitive development in adolescents. However, research examining their interrelationships remains scarce, and no nationwide survey studies targeting Chinese adolescents have been identified to date. Furthermore, findings on the combined effects of ABSI—a body composition indicator—and cardiorespiratory fitness on executive function remain scarce. To address this gap, this study conducted a cross-sectional assessment of cardiorespiratory fitness, ABSI, and executive function among 8,621 adolescents aged 13–18 across diverse regions of China. The aim is to analyze their interrelationships, thereby providing essential support for promoting both physical and cognitive health development among Chinese youth.

## 2 Methods

### 2.1 Participants

**Figure 1:**
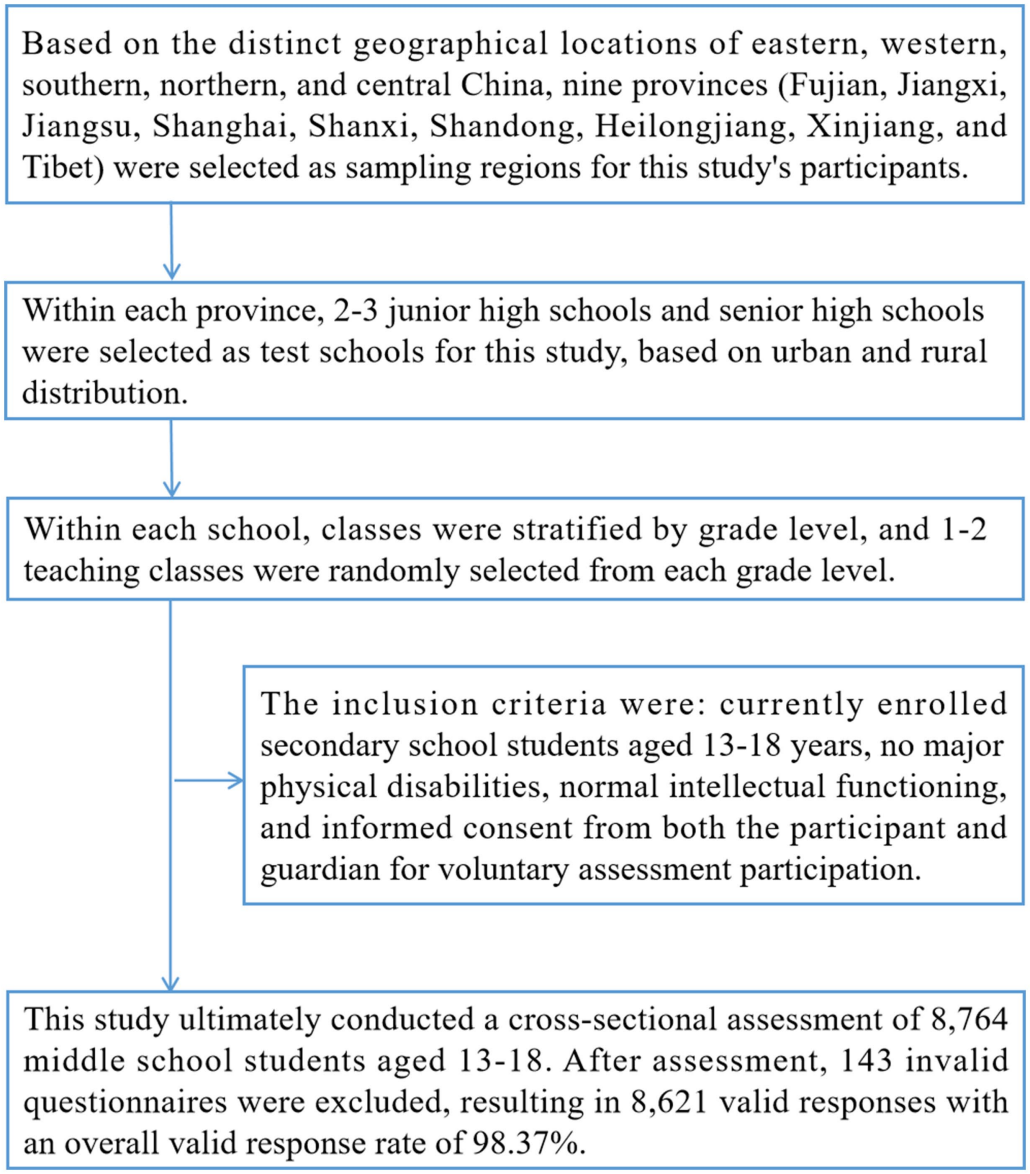
Sampling procedure for Chinese adolescent participants aged 13–18.

Participants were assessed during the period from 2022 to 2023. Participants in this study were randomly selected from nine regions in China using a three-stage stratified cluster sampling method. First, based on the distinct geographical locations of eastern, western, southern, northern, and central China, nine provinces (Fujian, Jiangxi, Jiangsu, Shanghai, Shanxi, Shandong, Heilongjiang, Xinjiang, and Tibet) were selected as sampling regions for this study’s participants. Second, within each province, 2–3 junior high schools and senior high schools were selected as test schools for this study, based on urban and rural distribution. Third, within each school, classes were stratified by grade level, and 1–2 teaching classes were randomly selected from each grade level. Based on the inclusion criteria of this study, eligible secondary school students within the class were enrolled for assessment. The inclusion criteria were: currently enrolled secondary school students aged 13–18 years, no major physical disabilities, normal intellectual functioning, and informed consent from both the participant and guardian for voluntary assessment participation. This study ultimately conducted a cross-sectional assessment of 8,764 middle school students aged 13–18, covering basic demographic information, lifestyle habits, ABSI, cardiorespiratory fitness, and executive function. After assessment, 143 invalid questionnaires were excluded, resulting in 8,621 valid responses with an overall valid response rate of 98.37%. The specific sampling process for participants is illustrated in Figure 1.

### 2.2 Executive function assessment

This study’s assessment of executive functions in Chinese adolescents primarily encompasses three core functions: inhibit control function, refreshing memory function, and switching flexibility function. (1) As a core component of executive function, inhibit control primarily assesses participants’ ability to suppress dominant responses, impulses, or external distractions. It also encompasses control over one’s emotions, attention, and behavioral habits, enabling better regulation or suppression of intense internal or external temptations. This facilitates the rational, reasonable, and correct execution of various tasks and abilities. (2) The refreshing memory function, as another core component of executive function, primarily involves dynamically replacing and modifying information currently maintained in working memory. This ensures the ability to rapidly process, manipulate, and retrieve memory content. (3) Switching flexibility function refers to the capacity to rapidly, accurately, and purposefully shift between multiple task rules, mental states, and stimulus dimensions.

In this study, the inhibition control function was assessed using the Flanker task paradigm. The memory refreshing function was evaluated using the 2-back task paradigm. The switching flexibility function was measured using the More-Odd Shifting task paradigm. All three functional paradigms are widely used internationally and demonstrate good reliability and validity for assessing executive function in adolescents (28). The specific evaluation methods for the three paradigms were detailed in previous studies (26). Prior to assessment, the three paradigms were installed on desktop computers. Staff provided participants with explanations and practice sessions for each paradigm before the formal evaluation. Testing commenced only after participants demonstrated full familiarity with each paradigm. Participants were required to complete the paradigm assessments in a quiet classroom with appropriate lighting and no external distractions. They were instructed to independently complete the evaluation tests for all three tasks. The assessment procedure was conducted using the E-prime 1.1 computer software system.

### 2.3 A body shape index assessment

ABSI primarily assesses abdominal obesity in participants. This metric has been validated in multiple studies to be more effective than BMI and WC in evaluating health risks. ABSI is calculated using BMI, WC, and height, with the specific formula being:


$$\text{ABSI}=\frac{\mathrm{WC}}{{\mathrm{BMI}}^{2/3}\times {\text{Height}}^{1/2}}$$

BMI is calculated as weight (kg) divided by height (m) squared, with units of kg/m^2^ (29). In this study, height, weight, and waist circumference (WC) were assessed according to the testing methods and standards specified by the China National Survey on Students’ Constitution and Health (CNSSCH) (30). All assessment instruments were calibrated daily prior to evaluation. Height was measured using the SZG-210 stadiometer. Weight was assessed using the TC-200 K weighing scale. Waist circumference (WC) was measured using a KMC-1600 nylon tape measure. Height and WC were recorded to the nearest 0.1 cm, while weight was recorded to the nearest 0.1 kg. Participants were instructed to empty their bowels and bladder prior to weight assessment. Additionally, participants were requested to wear lightweight clothing during measurements to ensure the accuracy of results.

### 2.4 VO_2max_ assessment

This study employed VO_2max_ to assess participants’ cardiorespiratory fitness levels (31). The assessment of VO_2max_ encompasses both direct and indirect evaluation methods. Indirect assessment methods, due to their simplicity and ease of implementation, are widely adopted for large-scale evaluations and have been extensively utilized in numerous studies. This research employs the 20-meter SRT to indirectly assess participants’ VO_2max_. The 20-meter SRT assessment method is currently used in over 50 countries worldwide to indirectly evaluate participants’ cardiorespiratory fitness levels (32). This study employed this method for assessment. Based on the assessment results, participants’ VO_2max_ was calculated using a fixed formula. Use Leger’s calculation equation to calculate VO_2max_ (33). The specific calculation equation is:


$$\begin{array}{l} {\mathrm{VO}}_{\mathrm{2max}}\;(\mathrm{mL}\cdot {\mathrm{kg}}^{-1}\cdot \min^{-1})=31.025+3.238\times S-3.248\times \\ \mathrm{Age}+0.1536\times S\times \mathrm{Age} \end{array}$$

S: the running speed at the last completed stage (km.h^−1^).

### 2.5 Covariates

Based on previous research data on factors affecting executive function, the covariates in this study primarily include dietary bias, SSB consumption, sleep quality, and community exercise frequency (34–36). Assessment was conducted using participant self-report questionnaires. Dietary bias was categorized as “Yes” or “No” based on participants’ responses to the evaluation questionnaire. SSB consumption was primarily determined by the frequency of sugar-sweetened beverage consumption over the past 7 days, with each serving calculated as a standard 330 mL/can (37). This study categorized participants into the following groups: 0 times/week, 1–2 times/week, 3–4 times/week, 5–6 times/week, and ≥7 times/week. Sleep quality was assessed primarily based on PSQI questionnaire scores, classified as Good (≤5 points), Medium (6–7 points), and Poor (>7 points) (38). Community exercise frequency is primarily categorized based on participants’ exercise habits over the past 7 days, divided into the following groups: 0 times/week, 1–2 times/week, 3–4 times/week, 5–6 times/week, and ≥7 times/week.

### 2.6 Statistical analysis

In this study, participants’ baseline characteristics were categorized into continuous variables and categorical variables. Continuous variables were expressed as mean ± standard deviation, while categorical variables were presented as percentages. Comparisons of baseline characteristics were conducted using t-tests and chi-square tests. ABSI and VO_2max_ (reflecting cardiorespiratory fitness) were stratified by age and sex and divided into quartiles: Q1, Q2, Q3, and Q4. Intergroup comparisons of participants’ different ABSI scores, cardiorespiratory fitness levels, and executive function responses were conducted using one-way analysis of variance (ANOVA). Based on previous studies, this research categorized participants by age and sex. Individuals whose reaction times for the inhibit control function, refreshing memory function, and switching flexibility function exceeded the mean by one standard deviation were defined as having inhibit control function dysfunction, refreshing memory function dysfunction, and switching flexibility function dysfunction, respectively (6). The detection rates of different ABSI levels, cardiorespiratory fitness, and executive function dysfunctions were compared using the chi-square test. The association between different ABSI scores, cardiorespiratory fitness, with executive function was analyzed using binary logistic regression. Binary logistic regression analyses were conducted with the presence of inhibit control function dysfunction, refreshing memory function dysfunction, and switching flexibility function dysfunction as dependent variables, and with ABSI and VO_2max_ as independent variables. Model 1 did not adjust for any covariates. Model 2 adjusted for age, dietary bias, SSB consumption, sleep quality, and community exercise frequency on top of Model 1. To further analyze the association between the combined effect of ABSI and VO_2max_ and executive function, this study conducted a binary logistic regression analysis using a generalized linear model. Model analysis controlled for age, dietary bias, SSB consumption, sleep quality, and community exercise frequency as covariates. Results are reported as odds ratios and 95% confidence intervals.

## 3 Results

This study conducted a cross-sectional assessment of basic demographic characteristics, ABSI, cardiorespiratory fitness, and executive function in 8,621 adolescents aged 13–18 years (4,407 boys, 51.12%). The mean age of participants was (15.14 ± 1.64) years. Overall, adolescent VO_2max_ was (41.12 ± 5.08) mL/kg/min, with boys exhibiting significantly higher VO_2max_ than girls (*t* = 24.617, *p <* 0.001). Overall, adolescents’ ABSI was (0.0615 ± 0.0240); boys demonstrated a statistically significant lower ABSI than girls (*t* = −3.384, *p <* 0.001). Overall, the detection rates for inhibit control dysfunction, refreshing memory dysfunction, and switching flexibility dysfunction among adolescents were 16.4, 19.7, and 16.1%, respectively. Comparing between sexes, boys exhibited significantly higher detection rates for refreshing memory dysfunction and switching flexibility dysfunction than girls (χ^2^ = 76.160, 8.408; *p <* 0.01). Sex comparisons for other items are shown in Table 1.

**Table 1:** Comparison of basic characteristics among chinese adolescents aged 13–18 by sex.

Items	Boys	Girls	*χ*^2^/*t*-value	*p*-value	Total
N	4,407	4,214			8,621
Age (years)	15.11 ± 1.66	15.16 ± 1.64	−1.580	0.114	15.14 ± 1.64
Height (M ± SD)	169.30 ± 8.80	162.23 ± 6.57	42.136	<0.001	165.85 ± 8.56
Weight (M ± SD)	62.48 ± 15.42	54.54 ± 11.44	27.053	<0.001	58.60 ± 14.19
BMI (M ± SD)	21.65 ± 4.46	20.67 ± 3.88	10.827	<0.001	21.17 ± 4.21
Waistline (M ± SD)	73.33 ± 29.41	69.12 ± 27.92	6.809	<0.001	71.27 ± 28.77
Inhibit control function reaction time (ms)	13.92 ± 11.83	13.45 ± 11.95	1.857	0.063	13.69 ± 11.89
Refreshing memory function reaction time (ms)	1050.54 ± 354.36	1059.77 ± 355.02	−1.209	0.227	1055.05 ± 354.69
Switching flexibility function reaction time (ms)	322.80 ± 180.75	317.73 ± 184.62	1.287	0.198	320.32 ± 182.66
20-m SRT (M ± SD)	45.01 ± 17.18	35.73 ± 12.12	28.841	<0.001	40.47 ± 15.63
VO_2max_ (M ± SD)	42.39 ± 5.20	39.79 ± 4.58	24.617	<0.001	41.12 ± 5.08
ABSI (M ± SD)	0.0607 ± 0.0234	0.0624 ± 0.0246	−3.384	0.001	0.0615 ± 0.0240
Dietary bias [N(%)]			112.216	<0.001	
Yes	1859(42.2)	2,258(53.6)			4,117(47.8)
No	2,548(57.8)	1956(46.4)			4,504(52.2)
SSB consumption [N(%)]			50.432	<0.001	
0 times/week	679(15.4)	563(13.4)			1,242(14.4)
1–2 times/week	1,663(37.7)	1782(42.3)			3,445(40)
3–4 times/week	1,277(29.0)	1,266(30.0)			2,543(29.5)
5–6 times/week	391(8.9)	363(8.6)			754(8.7)
≥7 times/week	397(9.0)	240(5.7)			637(7.4)
Sleep quality [N(%)]			93.516	<0.001	
Good	2,814(63.9)	2,261(53.7)			5,075(58.9)
Medium	862(19.6)	1,024(24.3)			1886(21.9)
Poor	731(16.6)	929(22.0)			1,660(19.3)
Community exercise frequency [N(%)]			170.461	<0.001	
0 times/week	2,341(53.1)	2,534(60.1)			4,875(56.5)
1–2 times/week	1,184(26.9)	1,220(29.0)			2,404(27.9)
3–4 times/week	478(10.8)	320(7.6)			798(9.3)
5–6 times/week	143(3.2)	70(1.7)			213(2.5)
≥7 times/week	261(5.9)	70(1.7)			331(3.8)
Inhibit control function dysfunction [N(%)]			0.063	0.802	
No	3,689(83.7)	3,519(83.5)			7,208(83.6)
Yes	718(16.3)	695(16.5)			1,413(16.4)
Refreshing memory function dysfunction [N(%)]			76.160	<0.001	
No	3,379(76.7)	3,546(84.1)			6,925(80.3)
Yes	1,028(23.3)	668(15.9)			1,696(19.7)
Switching flexibility function dysfunction [N(%)]			8.408	0.004	
No	3,648(82.8)	3,585(85.1)			7,233(83.9)
Yes	759(17.2)	629(14.9)			1,388(16.1)

N, numbers; M, Mean; SD, standard deviation; BMI, body mass index; SSB, sugar-sweetened beverage; 20-m SRT, 20-meter shuttle run test; ABSI, A body shape index.

Overall, the inhibit control function reaction time, the refreshing memory function reaction time, and the switching flexibility function reaction time of adolescents are (13.69 ± 11.89) ms, (1055.05 ± 354.69) ms, and (320.32 ± 182.66) ms, respectively. Compared with the reaction time between the ABSI (Q1, Q2, Q3, Q4) groups, the differences were also statistically significant (χ^2^ = 72.642, 184.613, 2826.466, *p* < 0.001). Similarly, compared with the VO_2max_ (Q1, Q2, Q3, Q4) groups, the differences were statistically significant (χ^2^ = 54.539, 47.333, 42.127, *p* < 0.001). The comparison results after sex stratification are shown in Table 2.

**Table 2:** Univariate analysis of variance of ABSI, cardiopulmonary fitness, and executive function reaction time in chinese adolescents aged 13–18 years.

Group	N	Inhibit control function reaction time (ms)			Refreshing memory function reaction time (ms)			Switching flexibility function reaction time (ms)		
		M (SD)	*F*-value	*p*-value	M (SD)	*F*-value	*p*-value	M (SD)	*F*-value	*p*-value
Boys										
ABSI			39.438	<0.001		99.223	<0.001		1316.923	<0.001
Q1	1,099	10.72 ± 11.32			919.42 ± 342.75			149.07 ± 144.64		
Q2	1,172	14.49 ± 11.99			1023.29 ± 348.51			269.66 ± 166.08		
Q3	1,179	15.76 ± 11.32			1115.88 ± 359.49			416.84 ± 114.88		
Q4	957	14.65 ± 12.12			1153.97 ± 314.86			471.53 ± 72.68		
VO_2max_			25.191	<0.001		30.68	<0.001		12.867	<0.001
Q1	805	16.54 ± 12.42			1128.49 ± 339.58			342.15 ± 181.42		
Q2	1,094	14.86 ± 11.41			1068.04 ± 355.61			337.77 ± 180.66		
Q3	1,046	12.82 ± 10.89			1061.58 ± 326.39			322.92 ± 182.53		
Q4	1,462	12.57 ± 12.16			986.62 ± 369.69			300.85 ± 176.93		
Girls										
ABSI			34.078	<0.001		85.894	<0.001		1545.793	<0.001
Q1	1,086	10.39 ± 10.96			942.72 ± 349.32			127.13 ± 149.23		
Q2	996	13.89 ± 11.91			1021.44 ± 381.91			273.16 ± 168.34		
Q3	964	14.74 ± 11.79			1108.47 ± 353.63			386.83 ± 102.95		
Q4	1,168	14.84 ± 12.48			1161.12 ± 296.26			475.93 ± 70.28		
VO_2max_			39.594	<0.001		17.872	<0.001		39.503	<0.001
Q1	1,441	15.45 ± 11.71			1096.44 ± 339.22			345.36 ± 172.10		
Q2	1,188	14.03 ± 12.59			1068.13 ± 361.60			334.25 ± 188.13		
Q3	902	12.22 ± 12.15			1052.38 ± 352.43			291.77 ± 185.56		
Q4	683	9.82 ± 9.87			977.64 ± 366.31			265.00 ± 187.50		
Total										
ABSI			72.642	<0.001		184.613	<0.001		2826.466	<0.001
Q1	2,185	10.55 ± 11.14			931.00 ± 346.15			138.17 ± 147.31		
Q2	2,168	14.21 ± 11.95			1022.44 ± 364.15			271.27 ± 167.09		
Q3	2,143	15.30 ± 11.54			1112.55 ± 356.80			403.34 ± 110.66		
Q4	2,125	14.75 ± 12.32			1157.90 ± 304.73			473.95 ± 71.38		
VO_2max_			54.539	<0.001		47.333	<0.001		42.127	<0.001
Q1	2,246	15.84 ± 11.98			1107.93 ± 339.62			344.21 ± 175.47		
Q2	2,282	14.43 ± 12.04			1068.09 ± 358.66			335.94 ± 184.56		
Q3	1948	12.54 ± 11.49			1057.32 ± 338.64			308.50 ± 184.54		
Q4	2,145	11.70 ± 11.55			983.76 ± 368.56			289.44 ± 181.09		

N, numbers; M, Mean; SD, standard deviation; ABSI, A body shape index.

Table 3 presents a comparison of the detection rates for ABSI, VO_2max_, and executive dysfunction among Chinese adolescents aged 13–18 years. Overall, statistically significant differences were observed in detection rates for inhibit control function dysfunction, refreshing memory function dysfunction, and switching flexibility function dysfunction across different ABSI groups (χ^2^ = 102.218, 169.837, 353.903; *p <* 0.001). Comparisons of detection rates for inhibit control dysfunction, refreshing memory dysfunction, and switching flexibility dysfunction among adolescents with different VO_2max_ levels also showed statistically significant differences (χ^2^ = 59.513, 110.335, 46.110, *p <* 0.001). Results stratified by sex are presented in Table 3.

**Table 3:** Comparative detection rates of ABSI, cardiopulmonary fitness, and executive function dysfunction among chinese adolescents aged 13–18 years.

Group	Number	Inhibit control function dysfunction			Refreshing memory function dysfunction			Switching flexibility function dysfunction		
		N(%)	*χ*^2^-value	*p*-value	N(%)	*χ*^2^-value	*p*-value	N(%)	*χ*^2^-value	*p*-value
Boys										
ABSI			58.975	<0.001		79.019	<0.001		237.104	<0.001
Q1	1,099	103(9.4)			165(15.0)			83(7.6)		
Q2	1,172	190(16.2)			254(21.7)			121(10.3)		
Q3	1,179	236(20.0)			352(29.9)			335(28.4)		
Q4	957	189(19.7)			257(26.9)			220(23.0)		
VO_2max_			23.672	<0.001		168.701	<0.001		34.691	<0.001
Q1	805	164(20.4)			307(38.1)			174(21.6)		
Q2	1,094	203(18.6)			299(27.3)			216(19.7)		
Q3	1,046	148(14.1)			186(17.8)			180(17.2)		
Q4	1,462	203(13.9)			236(16.1)			189(12.9)		
Girls										
ABSI			45.128	<0.001		96.665	<0.001		161.115	<0.001
Q1	1,086	110(10.1)			81(7.5)			79(7.3)		
Q2	996	175(17.6)			145(14.6)			100(10)		
Q3	964	178(18.5)			200(20.7)			160(16.6)		
Q4	1,168	232(19.9)			242(20.7)			290(24.8)		
VO_2max_			42.461	<0.001		24.675	<0.001		24.96	<0.001
Q1	1,441	294(20.4)			269(18.7)			238(16.5)		
Q2	1,188	205(17.3)			198(16.7)			208(17.5)		
Q3	902	130(14.4)			128(14.2)			113(12.5)		
Q4	683	66(9.7)			73(10.7)			70(10.2)		
Total										
ABSI			102.218	<0.001		169.837	<0.001		353.903	<0.001
Q1	1972	213(9.7)			246(11.3)			162(7.4)		
Q2	1803	365(16.8)			399(18.4)			221(10.2)		
Q3	1729	414(19.3)			552(25.8)			495(23.1)		
Q4	1704	421(19.8)			499(23.5)			510(24)		
VO_2max_			59.513	<0.001		110.335	<0.001		46.110	<0.001
Q1	2,246	458(20.4)			576(25.6)			412(18.3)		
Q2	2,282	408(17.9)			497(21.8)			424(18.6)		
Q3	1948	278(14.3)			314(16.1)			293(15.0)		
Q4	2,145	269(12.5)			309(14.4)			259(12.1)		

N, numbers; M, Mean; SD, standard deviation; ABSI, A body shape index.

Binary logistic regression analysis was conducted with the presence of inhibit control function dysfunction, refreshing memory function dysfunction, and switching flexibility function dysfunction as dependent variables, and ABSI and VO_2max_ as independent variables, respectively. Model 1 did not adjust for any covariates. Model 2 adjusted for age, dietary bias, SSB consumption, sleep quality, and community exercise frequency on top of Model 1. Overall results showed that compared with the ABSI Q1 group, the ABSI Q4 group had the highest risk of developing inhibit control function dysfunction (OR = 2.29, 95%CI: 1.91–2.73), refreshing memory function dysfunction (OR = 2.74, 95%CI: 2.31–3.25), and switching flexibility function dysfunction (OR = 2.87, 95%CI: 3.20–4.68; *p <* 0.001). Similarly, compared with the Q4 VO_2max_ group, the Q1 VO_2max_ group exhibited the highest risk of developing inhibit control dysfunction (OR = 2.01, 95%CI: 1.67–2.40), refreshing memory dysfunction (OR = 1.24, 95%CI:1.05–1.47), and switching flexibility dysfunction (OR = 2.10, 95%CI: 1.74–2.53; *p <* 0.001). Results from stratified by sex binary logistic regression analysis are presented in Table 4.

**Table 4:** Binary logistic regression analysis of ABSI, cardiopulmonary fitness, and executive function dysfunction among chinese adolescents aged 13–18 years.

Sex/variable	Group	Inhibit control function dysfunction				Refreshing memory function dysfunction				Switching flexibility function dysfunction			
		Model 1		Model 2		Model 1		Model 2		Model 1		Model 2	
		OR (95% CI)	*p-*value	OR (95% CI)	*p-*value	OR (95% CI)	*p-*value	OR (95% CI)	*p-*value	OR (95% CI)	*p-*value	OR (95% CI)	*p-*value
Boys													
ABSI	Q1	1.00		1.00		1.00		1.00		1.00		1.00	
	Q2	1.87(1.45–2.42)	<0.001	1.86(1.44–2.40)	<0.001	1.57(1.26–1.94)	<0.001	1.57(1.25–1.98)	<0.001	1.41(1.05–1.89)	0.021	1.39(1.04–1.87)	0.027
	Q3	2.42(1.89–3.10)	<0.001	2.39(1.86–3.07)	<0.001	2.41(1.96–2.96)	<0.001	2.70(2.16–3.39)	<0.001	4.86(3.76–6.28)	<0.001	4.75(3.66–6.15)	<0.001
	Q4	2.38(1.84–3.08)	<0.001	2.38(1.84–3.09)	<0.001	2.08(1.67–2.59)	<0.001	2.85(2.24–3.61)	<0.001	3.65(2.79–4.79)	<0.001	3.59(2.74–4.71)	<0.001
VO_2max_	Q4	1.00		1.00		1.00		1.00		1.00		1.00	
	Q3	1.02(0.81–1.29)	0.851	1.03(0.82–1.30)	0.792	1.12(0.91–1.39)	0.279	0.99(0.79–1.24)	0.942	1.40(1.12–1.75)	0.003	1.45(1.16–1.81)	0.001
	Q2	1.41(1.14–1.75)	0.001	1.46(1.17–1.82)	0.001	1.95(1.61–2.37)	<0.001	1.16(0.94–1.43)	0.176	1.66(1.34–2.05)	<0.001	1.82(1.46–2.27)	<0.001
	Q1	1.59(1.27–1.99)	<0.001	1.67(1.31–2.13)	<0.001	3.20(2.62–3.91)	<0.001	1.52(1.22–1.90)	<0.001	1.86(1.48–2.33)	<0.001	2.17(1.7–2.77)	<0.001
Girls													
ABSI	Q1	1.00		1.00		1.00		1.00		1.00		1.00	
	Q2	1.89(1.46–2.44)	<0.001	1.89(1.46–2.45)	<0.001	2.11(1.59–2.82)	<0.001	2.11(1.58–2.81)	<0.001	1.42(1.05–1.94)	0.025	1.43(1.05–1.95)	0.024
	Q3	2.01(1.56–2.60)	<0.001	2.00(1.55–2.58)	<0.001	3.25(2.47–4.28)	<0.001	3.29(2.50–4.34)	<0.001	2.54(1.91–3.37)	<0.001	2.49(1.87–3.32)	<0.001
	Q4	2.20(1.72–2.81)	<0.001	2.20(1.72–2.81)	<0.001	3.24(2.48–4.24)	<0.001	3.27(2.50–4.28)	<0.001	4.21(3.23–5.49)	<0.001	4.15(3.18–5.42)	<0.001
VO_2max_	Q4	1.00		1.00		1.00		1.00		1.00		1.00	
	Q3	1.57(1.15–2.16)	0.005	1.67(1.21–2.29)	0.002	1.38(1.02–1.88)	0.039	1.48(1.09–2.02)	0.012	1.25(0.91–1.72)	0.160	1.41(1.02–1.94)	0.036
	Q2	1.95(1.45–2.62)	<0.001	2.14(1.59–2.89)	<0.001	1.67(1.26–2.23)	<0.001	1.83(1.37–2.45)	<0.001	1.86(1.39–2.48)	<0.001	2.17(1.62–2.92)	<0.001
	Q1	2.40(1.80–3.18)	<0.001	3.10(2.26–4.25)	<0.001	1.92(1.46–2.53)	<0.001	2.43(1.78–3.31)	<0.001	1.73(1.31–2.30)	<0.001	2.64(1.92–3.63)	<0.001
Total													
ABSI	Q1	1.00		1.00		1.00		1.00		1.00		1.00	
	Q2	1.87(1.56–2.25)	<0.001	1.87(1.56–2.24)	<0.001	1.78(1.50–2.11)	<0.001	1.73(1.46–2.06)	<0.001	1.42(1.15–1.75)	0.001	1.40(1.13–1.73)	0.002
	Q3	2.22(1.86–2.65)	<0.001	2.20(1.84–2.63)	<0.001	2.74(2.32–3.22)	<0.001	2.94(2.48–3.48)	<0.001	3.75(3.11–4.53)	<0.001	3.67(3.04–4.44)	<0.001
	Q4	2.29(1.92–2.73)	<0.001	2.29(1.91–2.73)	<0.001	2.42(2.05–2.86)	<0.001	2.74(2.31–3.25)	<0.001	3.94(3.27–4.76)	<0.001	3.87(3.20–4.68)	<0.001
VO_2max_	Q4	1.00		1.00		1.00		1.00		1.00		1.00	
	Q3	1.16(0.97–1.39)	0.104	1.19(0.99–1.42)	0.066	1.14(0.96–1.35)	0.128	1.06(0.90–1.27)	0.483	1.29(1.08–1.54)	0.006	1.37(1.14–1.64)	0.001
	Q2	1.52(1.29–1.79)	<0.001	1.60(1.35–1.90)	<0.001	1.65(1.42–1.93)	<0.001	1.30(1.10–1.53)	0.002	1.66(1.41–1.96)	<0.001	1.87(1.57–2.21)	<0.001
	Q1	1.79(1.52–2.11)	<0.001	2.01(1.67–2.40)	<0.001	2.05(1.76–2.39)	<0.001	1.24(1.05–1.47)	0.013	1.64(1.38–1.94)	<0.001	2.10(1.74–2.53)	<0.001

OR, Odds Ratio; 95% CI, 95% Confidence Interval.

**Figure 2:**
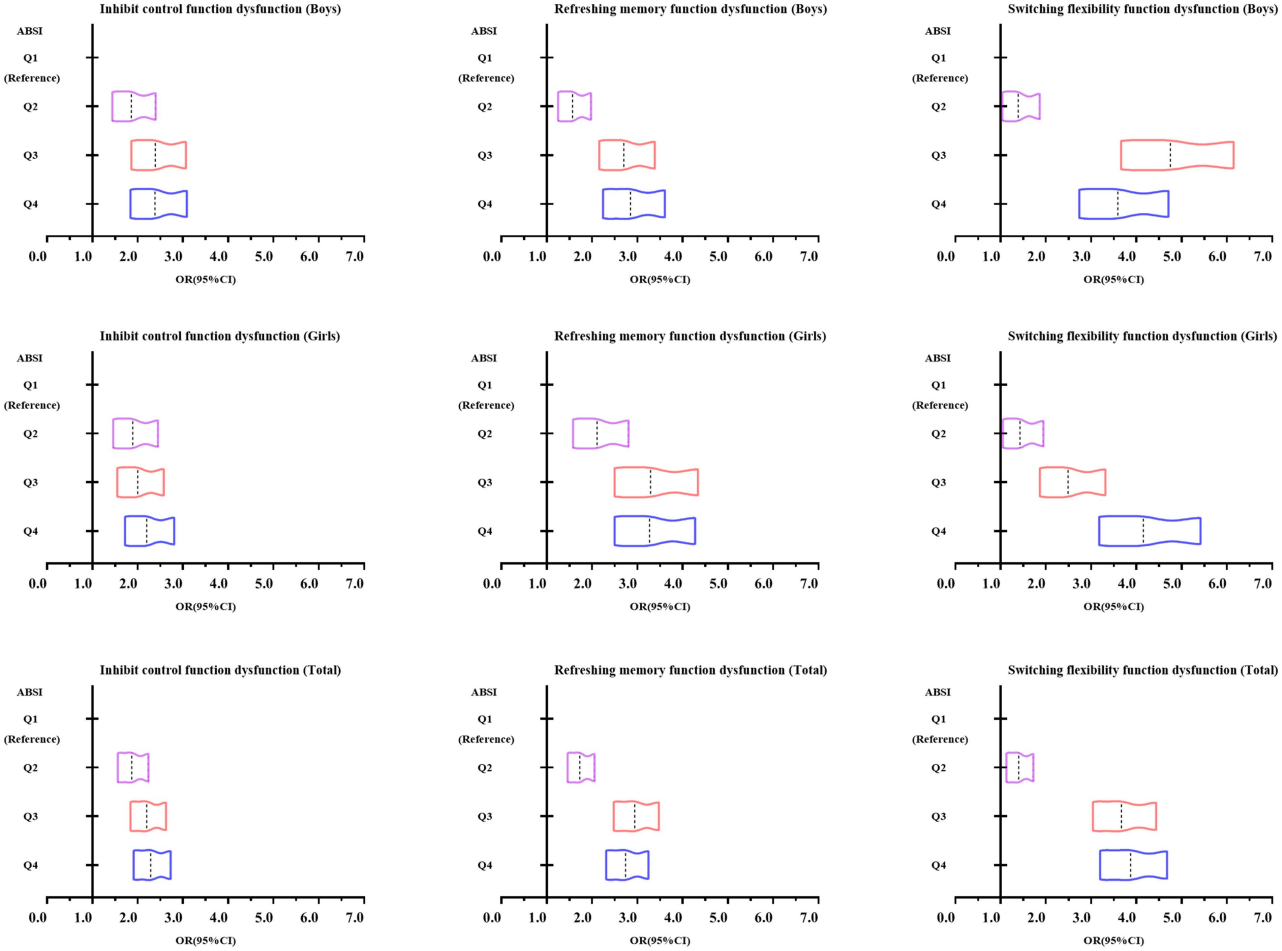
Trends in odds ratio values from binary logistic regression analysis of ABSI and executive function dysfunction among Chinese adolescents aged 13–18 years.

Figure 2 shows the trend in odds ratio values from a binary logistic regression analysis examining the association between ABSI and executive dysfunction among Chinese adolescents aged 13–18 years. The figure indicates that as ABSI increases, the risk of executive dysfunction progressively rises, as evidenced by the rightward shift in the OR values.

**Figure 3:**
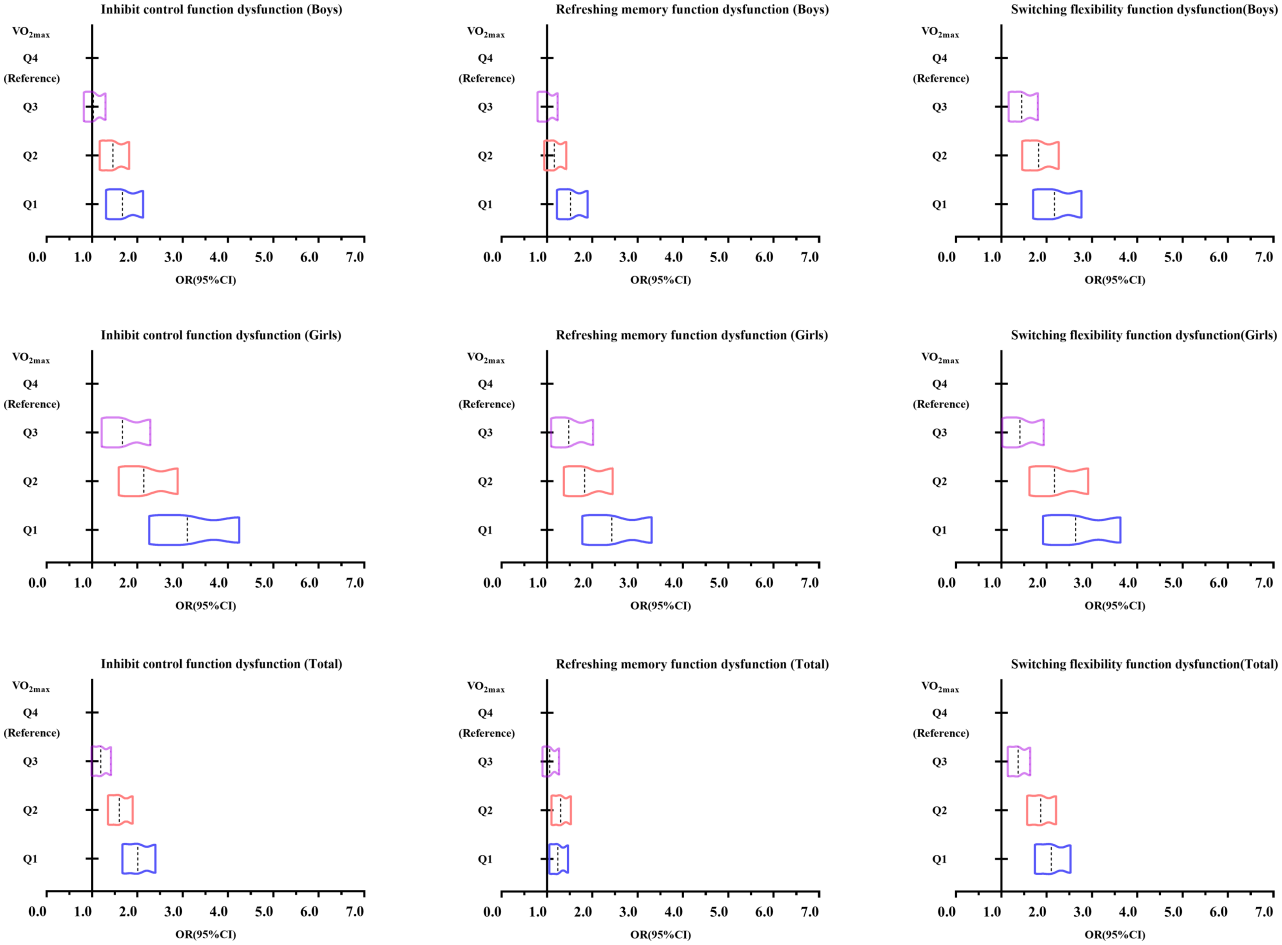
Trends in odds ratio values from binary logistic regression analysis of cardiopulmonary fitness and executive function dysfunction among Chinese adolescents aged 13–18 years.

Figure 3 shows the trend in odds ratio values from a binary logistic regression analysis examining the association between cardiorespiratory fitness and executive dysfunction in Chinese adolescents aged 13–18 years. The figure indicates that as cardiorespiratory fitness declines, the risk of executive dysfunction increases progressively, as evidenced by the rightward shift in the odds ratio values.

To further analyze the association between the joint effects of VO_2max_ and ABSI and executive dysfunction, this study employed binary logistic regression analysis using generalized linear models. The dependent variables were categorized as the presence or absence of inhibit control dysfunction, refreshing memory dysfunction, and switching flexibility dysfunction. The independent variables comprised the interaction groups of ABSI quartiles and VO_2max_ quartiles. Model analyses controlled for age, dietary bias, SSB consumption, sleep quality, and community exercise frequency as covariates. Overall results indicate that compared to the group with ABSI in Q1 and VO_2max_ in Q4, the group with ABSI in Q3 and VO_2max_ in Q1 exhibited the highest risk of developing inhibit control function dysfunction (OR = 3.49, 95%CI: 2.52–4.85), refreshing memory function dysfunction (OR = 6.20, 95%CI: 4.42–8.70), and switching flexibility function dysfunction (OR = 4.76, 95%CI: 3.48–6.52; *p <* 0.001). Analysis results stratified by sex are presented in Table 5.

**Table 5:** Binary logistic regression analysis using generalized linear models for ABSI, cardiopulmonary fitness, and executive function dysfunction among chinese adolescents aged 13–18 years.

Sex	Classification of interaction		Inhibit control function dysfunction		Refreshing memory function dysfunction		Switching flexibility function dysfunction	
	ABSI	VO_2max_	OR (95% CI)	*p*-value	OR (95% CI)	*p*-value	OR (95% CI)	*p*-value
Boys	Q1	Q4	1.000		1.000		1.000	
		Q3	0.56(0.31–1.02)	0.058	0.89(0.51–1.55)	0.677	0.92(0.52–1.64)	0.787
		Q2	0.92(0.55–1.56)	0.765	3.74(2.41–5.79)	<0.001	0.93(0.52–1.66)	0.796
		Q1	1.37(0.78–2.41)	0.28	3.81(2.33–6.25)	<0.001	0.82(0.40–1.71)	0.598
	Q2	Q4	1.26(0.81–1.95)	0.309	1.63(1.04–2.56)	0.033	1.05(0.63–1.74)	0.855
		Q3	1.64(1.04–2.59)	0.033	2.05(1.29–3.27)	0.003	1.34(0.79–2.26)	0.28
		Q2	2.27(1.48–3.48)	<0.001	3.29(2.13–5.07)	<0.001	1.51(0.91–2.49)	0.112
		Q1	1.93(1.22–3.03)	0.005	6.22(4.07–9.52)	<0.001	1.53(0.91–2.58)	0.111
	Q3	Q4	1.59(1.04–2.44)	0.034	3.19(2.10–4.84)	<0.001	2.24(1.43–3.51)	<0.001
		Q3	1.95(1.24–3.05)	0.004	3.72(2.40–5.76)	<0.001	4.68(3.00–7.28)	<0.001
		Q2	2.55(1.68–3.87)	<0.001	4.35(2.86–6.62)	<0.001	5.41(3.53–8.29)	<0.001
		Q1	3.10(2.04–4.70)	<0.001	7.81(5.16–11.84)	<0.001	7.69(5.02–11.79)	<0.001
	Q4	Q4	2.13(1.40–3.25)	<0.001	2.84(1.84–4.37)	<0.001	3.11(2.00–4.85)	<0.001
		Q3	1.88(1.19–2.96)	0.006	2.86(1.82–4.49)	<0.001	3.44(2.18–5.43)	<0.001
		Q2	2.40(1.55–3.71)	<0.001	4.48(2.91–6.89)	<0.001	4.20(2.69–6.57)	<0.001
		Q1	2.50(1.51–4.16)	<0.001	8.00(4.98–12.85)	<0.001	2.85(1.66–4.90)	<0.001
Girls	Q1	Q4	1.000		1.000		1.000	
		Q3	1.81(0.92–3.57)	0.085	0.88(0.37–2.11)	0.777	0.65(0.35–1.22)	0.18
		Q2	2.05(1.06–3.98)	0.033	2.55(1.24–5.22)	0.011	0.85(0.48–1.53)	0.597
		Q1	3.29(1.75–6.18)	<0.001	3.09(1.52–6.27)	0.002	0.39(0.19–0.80)	0.011
	Q2	Q4	1.81(0.84–3.91)	0.13	3.69(1.73–7.88)	0.001	0.78(0.38–1.60)	0.506
		Q3	3.20(1.65–6.19)	0.001	4.12(2.02–8.42)	<0.001	0.67(0.34–1.33)	0.254
		Q2	4.15(2.23–7.72)	<0.001	3.62(1.80–7.27)	<0.001	1.35(0.78–2.32)	0.281
		Q1	4.84(2.68–8.76)	<0.001	4.12(2.12–8.03)	<0.001	1.09(0.65–1.84)	0.738
	Q3	Q4	2.27(1.06–4.86)	0.035	4.56(2.14–9.72)	<0.001	0.51(0.21–1.20)	0.124
		Q3	4.59(2.44–8.65)	<0.001	4.78(2.37–9.63)	<0.001	0.99(0.54–1.82)	0.976
		Q2	3.17(1.68–5.99)	<0.001	6.59(3.37–12.87)	<0.001	1.92(1.15–3.22)	0.013
		Q1	5.19(2.86–9.39)	<0.001	7.01(3.65–13.46)	<0.001	2.97(1.85–4.76)	<0.001
	Q4	Q4	3.70(1.84–7.46)	<0.001	3.80(1.75–8.24)	0.001	2.10(1.16–3.80)	0.014
		Q3	2.94(1.52–5.71)	0.001	7.10(3.59–14.04)	<0.001	3.61(2.18–5.98)	<0.001
		Q2	5.28(2.93–9.52)	<0.001	5.71(2.97–10.99)	<0.001	3.49(2.19–5.54)	<0.001
		Q1	4.72(2.63–8.47)	<0.001	6.50(3.41–12.37)	<0.001	2.69(1.69–4.26)	<0.001
Total	Q1	Q4	1.000		1.000		1.000	
		Q3	0.91(0.6–1.38)	0.662	0.81(0.51–1.30)	0.385	0.79(0.52–1.21)	0.284
		Q2	1.22(0.82–1.80)	0.327	2.88(1.99–4.16)	<0.001	0.91(0.61–1.38)	0.668
		Q1	1.92(1.31–2.81)	0.001	2.76(1.87–4.07)	<0.001	0.55(0.33–0.92)	0.023
	Q2	Q4	1.44(0.98–2.10)	0.063	2.10(1.43–3.08)	<0.001	0.94(0.62–1.41)	0.764
		Q3	2.02(1.4–2.92)	<0.001	2.52(1.72–3.69)	<0.001	1.02(0.68–1.54)	0.919
		Q2	2.71(1.92–3.82)	<0.001	3.11(2.17–4.48)	<0.001	1.45(1.01–2.10)	0.046
		Q1	2.76(1.98–3.86)	<0.001	4.21(2.98–5.95)	<0.001	1.31(0.91–1.88)	0.149
	Q3	Q4	1.82(1.26–2.64)	0.002	3.65(2.54–5.26)	<0.001	1.61(1.11–2.33)	0.012
		Q3	2.62(1.84–3.74)	<0.001	3.78(2.63–5.45)	<0.001	2.65(1.87–3.75)	<0.001
		Q2	2.59(1.84–3.65)	<0.001	4.70(3.31–6.65)	<0.001	3.52(2.54–4.88)	<0.001
		Q1	3.49(2.52–4.85)	<0.001	6.20(4.42–8.70)	<0.001	4.76(3.48–6.52)	<0.001
	Q4	Q4	2.55(1.78–3.66)	<0.001	3.16(2.17–4.61)	<0.001	2.70(1.90–3.84)	<0.001
		Q3	2.10(1.46–3.04)	<0.001	3.86(2.68–5.56)	<0.001	3.58(2.55–5.01)	<0.001
		Q2	3.16(2.27–4.40)	<0.001	4.20(2.97–5.94)	<0.001	3.93(2.86–5.40)	<0.001
		Q1	2.98(2.13–4.16)	<0.001	4.97(3.52–7.02)	<0.001	2.96(2.13–4.11)	<0.001

OR, Odds Ratio; 95% CI, 95% Confidence Interval.

**Figure 4:**
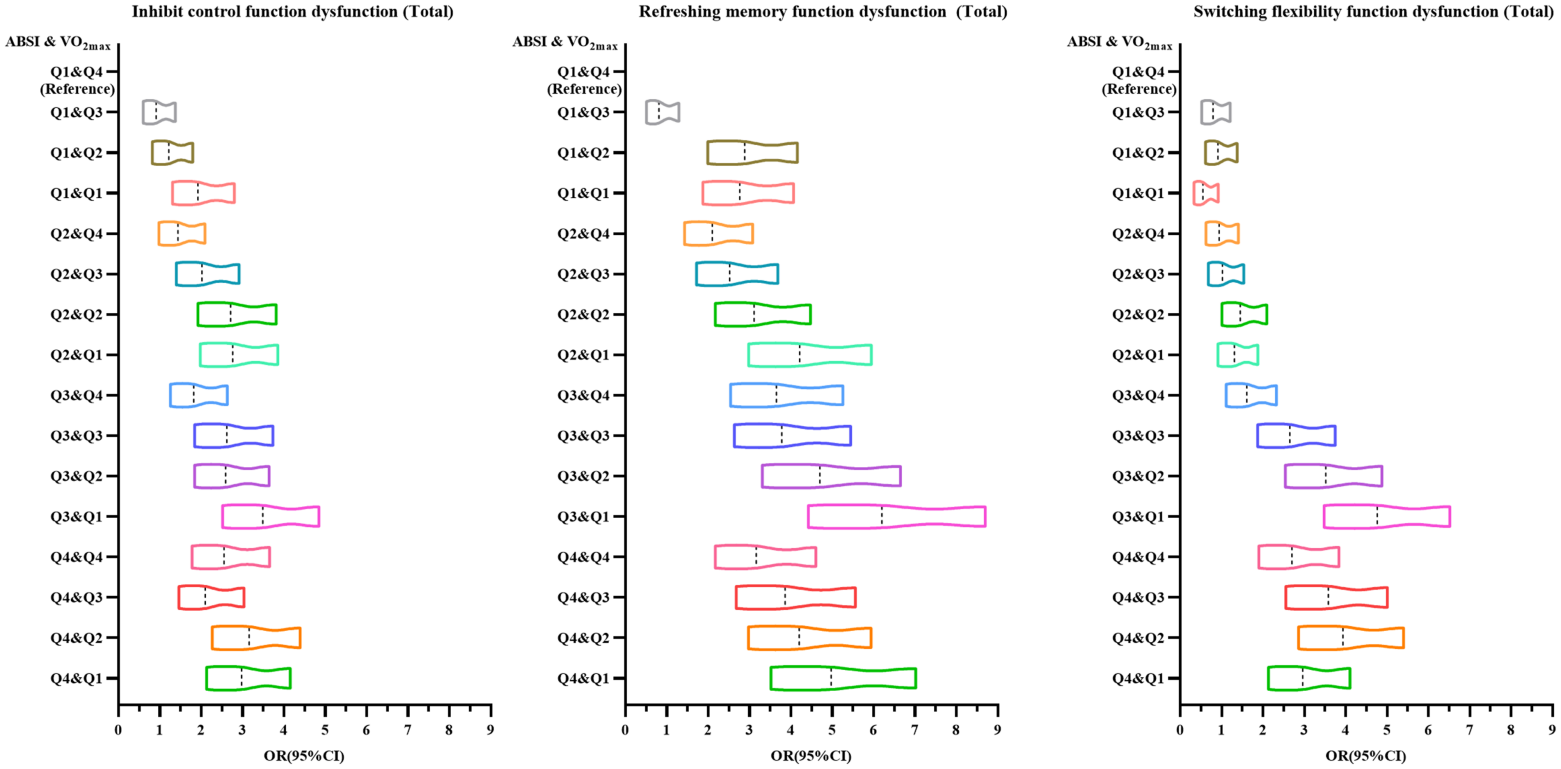
Trend in odds ratio values from binary logistic regression analysis using generalized linear models for ABSI, cardiopulmonary fitness, and executive function dysfunction among Chinese adolescents aged 13–18 years.

Figure 4 shows the trend in odds ratio values from a generalized linear model binary logistic regression analysis examining the association between ABSI, cardiorespiratory fitness, and executive dysfunction among Chinese adolescents aged 13–18 years. The figure indicates that as ABSI increases and cardiorespiratory fitness decreases, the risk of executive dysfunction among adolescents rises, with OR values shifting progressively to the right.

## 4 Discussion

Executive functions exert a significant influence on adolescents’ academic performance and future achievements, attracting considerable scholarly attention in recent years (39). Research indicates that adolescents’ executive function levels not only effectively predict academic performance but also significantly influence their emotional state, social behavior, and mental health (40). This demonstrates that in-depth analysis of factors influencing adolescents’ executive function development holds significant practical implications for enhancing their executive function levels. The findings of this study indicate that the reaction times for inhibit control function, refreshing memory function, and switching flexibility function among Chinese adolescents were (13.69 ± 11.89) ms, (1055.05 ± 354.69) ms, and (320.32 ± 182.66) ms, respectively. These results are consistent with those from relevant domestic studies (41). However, compared with studies on Tibetan adolescents in high-altitude regions, the results of this study indicate relatively shorter executive function reaction times. In high-altitude regions, the oxygen-deprived environment of the plateau leads to lower levels of executive function in the brain. The findings of this study also indicate that there are no significant differences in executive function levels among adolescents based on sex. This result is consistent with the conclusions of related research (42). The results of this study indicate that Chinese adolescent boys exhibit a statistically significantly higher VO_2max_ than girls. On one hand, influenced by genetic factors, boys possess greater muscular strength than girls, leading to superior performance in cardiorespiratory fitness assessments (43). Boys typically engage in higher levels of physical activity than girls, with greater intensity and frequency in their participation in sports and exercise. These factors are key reasons why boys exhibit higher VO_2max_ values than girls (44). Regarding ABSI, the findings of this study indicate that Chinese adolescent girls exhibit significantly higher ABSI scores than boys, consistent with the conclusions of multiple studies (45, 46). This outcome is associated with developmental imbalances between sexes during adolescence. Girls primarily gain fat during puberty, particularly in the form of central obesity, while boys experience predominant height growth. This leads to significant sex differences in ABSI levels (47).

ABSI, as a novel indicator comprehensively reflecting adolescent body composition, demonstrates significant advantages over metrics such as BMI and WC in predicting all-cause mortality risk and the onset of various chronic diseases (48). However, past research has rarely examined the association between ABSI and executive function. The findings of this study indicate that adolescents with ABSI in the Q4 group exhibited longer reaction times for inhibit control function, refreshing memory function, and switching flexibility function compared to those in the Q1 group, reflecting poorer executive function levels. Multiple factors contributed to this outcome. First, a higher ABSI in adolescents, indicative of greater abdominal adiposity, is linked to impaired executive function. This association is mediated through a cascade where increased fat mass triggers systemic inflammation, leading to mitochondrial damage and reduced synaptic plasticity in the prefrontal cortex (49). Second, Increased body fat contributes to insulin resistance and elevated cortisol, which in turn reduces norepinephrine and dopamine secretion in the prefrontal cortex. This neurochemical deficit ultimately impairs the core components of executive function, including attention, inhibit control, and cognitive flexibility (50). Third, increased fat intake leads to certain changes in the gut microbiota of adolescents, which in turn alters the gut-brain axis and affects executive function levels (51). Additionally, increased body fat elevates the risk of atherosclerosis, impairing blood flow and reducing its volume. The prefrontal cortex is most sensitive to decreases in cerebral blood flow, thereby adversely affecting executive function to a certain extent (52). In addition to being influenced by body fat levels, adolescents’ executive function is also affected by cardiorespiratory fitness levels.

Cardiorespiratory fitness, as a core component of adolescents’ physical health, holds significant practical importance for their physical and mental development. VO_2max_ is commonly used as an indicator to indirectly reflect adolescents’ cardiorespiratory fitness levels (53). The findings of this study indicate that adolescents with lower cardiorespiratory fitness exhibit relatively lower levels of executive function compared to those with higher cardiorespiratory fitness, revealing a negative correlation between the two. The reasons for this association may be attributed to several factors. First, individuals with high cardiorespiratory fitness exhibit greater stroke volume, increasing cerebral blood flow, and elevating perfusion pressure in the prefrontal cortex. Over time, this promotes increased capillary density, ensuring ample energy supply to the executive network. Consequently, this enhances the brain’s reaction speed and executive function levels (54). Research indicates that individuals with higher cardiorespiratory fitness exhibit greater cerebral blood flow and enhanced oxygen delivery to the brain, positively influencing brain activation levels (55). Secondly, individuals with higher cardiorespiratory fitness experience elevated levels of lactate and ketone bodies during moderate-to-high intensity exercise. This triggers the CREB-BDNF pathway, leading to increased synthesis of synaptic proteins in the hippocampus and prefrontal cortex, along with enhanced dendritic spine density. This process provides new neuronal and synaptic resources for executive function, thereby promoting improvements in executive function performance (56, 57). Third, individuals with higher cardiorespiratory fitness are better able to suppress the release of visceral fat inflammatory mediators (IL-6, TNF-*α*), reduce neuroinflammation, and more effectively enhance executive function levels (57). This study further analyzed the combined effects of different combinations of ABSI and cardiorespiratory fitness on three sub-functions of executive function. The findings reveal that, overall, the interaction between ABSI and cardiorespiratory fitness exerts a more pronounced effect on adolescents’ refreshing memory function. However, it is noteworthy that as ABSI increases in adolescents (Q3, Q4), the impact of cardiorespiratory fitness on their switching flexibility function rises sharply. This indicates that increased abdominal fat is more sensitive to switching flexibility function.

This study possesses certain strengths and limitations. Regarding strengths: First, to our knowledge, this study represents the first nationwide analysis using a large sample to examine the associations among ABSI, cardiorespiratory fitness, and executive function among Chinese adolescents. It provides a theoretical basis for enhancing and promoting executive function levels among Chinese youth, demonstrating considerable representativeness. Second, this study employed computer software to assess adolescents’ executive function levels, yielding relatively objective and accurate results. However, certain limitations exist. First, this is a cross-sectional study. It can only analyze the cross-sectional associations between adolescents’ ABSI, cardiorespiratory fitness, and executive function, without establishing causal relationships. Future prospective cohort studies are needed to examine this causal relationship. Second, the number of covariates included in the analysis was limited. Future studies should incorporate additional variables influencing adolescent executive function, such as physical activity, smoking, alcohol consumption, and family economic status, to achieve more precise analytical results.

## 5 Conclusion

Chinese adolescents exhibit both independent and joint associations between body fat percentage, cardiorespiratory fitness, and three sub-functions of executive function, with no sex differences observed in these relationships. Effectively controlling body fat levels and enhancing cardiorespiratory fitness in Chinese adolescents plays a significant positive role in promoting the development of their executive function. Future interventions aimed at enhancing adolescents’ executive function should incorporate body fat percentage and cardiorespiratory fitness as key reference indicators within their intervention strategies. This approach will better promote the development of executive function in Chinese adolescents, thereby improving academic performance and future achievements.

## Data Availability

The raw data supporting the conclusions of this article will be made available by the authors, without undue reservation.
